# Isolation of Candidatus Bartonella melophagi from Human Blood^1^

**DOI:** 10.3201/eid1501.081080

**Published:** 2009-01

**Authors:** Ricardo G. Maggi, Michael Kosoy, Melanie Mintzer, Edward B. Breitschwerdt

**Affiliations:** North Carolina State University, Raleigh, North Carolina, USA (R.G. Maggi and E.B. Breitschwerdt); Centers for Disease Control and Prevention, Fort Collins, Colorado, USA (M. Kosoy); Generations Family Practice, Cary, North Carolina, USA (M. Mintzer)

**Keywords:** Bacteria, Bartonella, sheep, keds, B. melophagi, B. henselae, blood-borne infection, dispatch

## Abstract

Candidatus *Bartonella melophagi* was isolated by blood culture from 2 women, 1 of whom was co-infected with *B*. *henselae*. Partial 16S rRNA, RNA polymerase B, and citrate synthase genes and 16S–23S internal transcribed spacer sequences indicated that human isolates were similar to Candidatus *B. melophagi*.

During the past decade, the number of *Bartonella* species that are documented human pathogens has rapidly increased (*1*). Currently, *B*. *bacilliformis*, *B*. *quintana*, *B*. *henselae*, *B*. *elizabethae*, *B*. *vinsonii* subsp. *berkhoffii*, *B*. *vinsonii* subsp. *arupensis*, *B*. *koehlerae*, *B*. *alsatica*, *B*. *washoensis*, *B*. *rochalimae*, and *B*. *tamiae* have been isolated or sequenced from patient samples (*1*–*7*). Sheep are the most likely reservoir hosts for Candidatus *B*. *melophagi* and sheep keds may be a vector for their transmission among sheep. We report isolation of Candidatus *B*. *melophagi* from blood cultures from 2 women. This study was reviewed and approved by the North Carolina State University Institutional Review Board.

## The Study

Patient 1 was a previously healthy, 51-year-old woman. During July 2004, she visited family residing in rural Ohio and participated in a variety of outdoor activities. Although she saw many wild animals, including deer, she did not report tick attachment or insect bites. Within 24 hours of her return home to North Carolina, a nonpuritic, slightly raised, circular red lesion, approximately the size of a quarter, was noted on the medial aspect of her thigh. Within 3 days, the lesion expanded to the size of a hand. Two weeks later, she exhibited a dry cough, fatigue, muscle pain in the upper body, severe chills, and extreme pain in both feet.

During the next 2 years, these symptoms persisted, along with exertional chest pains, a previously undiagnosed ausculted II to III/VI holosystolic murmur, headaches, difficulty speaking, difficulty sleeping, weakness involving the arms, joint pain, and facial tremors. No abnormalities were shown on an electrocardiogram. An echocardiogram identified mildly thickened aortic and mitral valve leaflets, mild aortic insufficiency, and mild mitral regurgitation.

After the acute illness, the woman reported cycles of illness every 3 to 4 weeks. Results of numerous complete blood counts were normal, with the exception of persistently low neutrophil counts of 2,000–2,500 neutrophils/μL. All serum biochemical parameters remained within normal reference ranges during the 2-year illness. *Borrelia burgdorferi* C6 peptide and immunoglobulin (Ig) M and IgG antibodies to *Babesia microti* were not detected. Results of PCRs specific for *Anaplasma phagocytophilum*, *B*. *microti*, and *B*. *burgdorferi* were negative. Oral antimicrobial drugs resulted in transient improvement; however, symptoms returned within days after the use of these drugs was stopped. Blood culture resulted in the detection of Candidatus *B*. *melophagi* and isolation of *B*. *henselae*. Her serum was not reactive with *B*. *henselae* or *B*. *vinsonii* subsp. *berkhoffii* antigens.

Treatment with rifampin and azithromycin, started in January 2006, resulted in some overall improvement in symptoms. Cefuroxime was added in February, and the combination resulted in substantial improvement, after which the drugs were selectively withdrawn. For 15 years before the onset of illness, this person had worked as an animal shelter manager in West Virginia and as a veterinary office manager in Virginia. Animal contact was minimal, but she had been bitten by fleas and mosquitoes. Travel history was limited to the eastern and central United States.

Patient 2 was a 65-year-old woman whose condition had been diagnosed as pericarditis of undetermined etiology in September 2004. Six months later, because of residual fatigue and muscle weakness in the arms and legs, mostly on her right side, a blood sample was cultured in *Bartonella* alpha proteobacteria growth medium (BAPGM).

The woman lived on a farm in southern California with her husband and managed a large animal sanctuary that also housed ≈100 cats and ≈100 dogs. She had resided in southern California for 50 years but occasionally traveled to the southeastern United States and other countries. She was directly involved in daily care of animals and had exposure to pet cattle and sheep, wolf hybrids, lamas, emus, pigs, horses, and numerous pet bird species. Bites and scratches were a daily occurrence, and exposure to cattle and sheep occurred at least weekly. In addition, the woman reported daily exposure to biting flies, occasional exposure to ticks and mosquitoes, and infrequent exposure to fleas or lice. Sheep keds had never been observed on sheep by the attending veterinarian. Blood culture resulted in isolation of Candidatus *B*. *melophagi*. Serum was reactive at a titer of 64 to *B*. *henselae*, *B*. *vinsonii* subsp. *berkhoffii*, and *B*. *quintana* antigens.

We used BAPGM and other published blood culture methods to test blood samples from both women (*2*,*8*,*9*). Candidatus *B*. *melophagi* DNA was amplified directly from blood of patient 2, and from the respective BAPGM enrichment cultures and 14-day subculture colonies from both patients. Sequence analysis of respective colony isolates showed *B*. *henselae* (internal transcribed spacer [ITS] sequence identical to Houston 1 strain, data not shown) and Candidatus *B*. *melophagi* from patient 1 and Candidatus *B*. *melophagi* (isolate 05-HO-1) from patient 2. Both isolates were composed of extremely small gram-negative bacilli consistent with *Bartonella* spp. Sequence analyses for both isolates are summarized in the Table. Unfortunately, attempts to separate *B*. *henselae* and Candidatus *B*. *melophagi* colonies from the sample of patient 1 by serial passage were unsuccessful. *Bartonella* sp. DNA was not amplified from an uninoculated BAPGM control culture or from sheep blood used as a supplement. Flagella, as visualized in the Candidatus *B*. *melophagi* strain K-2C isolated from sheep blood (Figure), were not visualized in the human 05-HO-2 strain by transmission electron microscopy.

**Table:** Sequence similarities for 16S–23S ITS and 3 genes from 2 patient isolates and available GenBank sequences*

Sequence or gene	Basepair homology (%)	Basepair homology (%)
ITS	*Bartonella* sp. tick†	*Bartonella melophagi‡*
Patient 1	405/408 (99.3)	385/388 (99.2)
Patient 2	405/408 (99.3)	385/388 (99.2)
*gltA*	*Bartonella* sp. sheep blood§	*B. melophagi*¶
Patient 1	131/134 (97.8)	183/187 (97.9)
*rpoB*		*B. melophagi#*
Patient 1	NA	651/656 (99.2)
16S rRNA	Wolbachia melophagi**	*B. melophagi*††
Patient 1	670/671 (99.8)	631/633 (99.7)

*ITS, internal transcribed spacer; gltA, citrate synthase; *rpoB*, RNA polymerase B; NA, not available. †Uncultured *Bartonella* sp. clone BT7498 sequenced from a tick from Peru (GenBank accession no. AF415209). ‡Candidatus *B. melophagi* strain K-2C isolated from a sheep ked (M. Kosoy, unpub. data). §*Bartonella* sp. isolated from commercial sheep blood agar plates (GenBank accession no. EU020109). ¶Candidatus *B. melophagi* strains K-9B and K-2C isolated from sheep ked (GenBank accession nos. AY724769 and AY724769). #Candidatus *B. melophagi* strain K-2C isolated from sheep ked (GenBank accession no. EF605288). **W. melophagi sequenced from *Melophagus ovinus* sheep keds (GenBank accession no. X89110). ††Candidatus *B. melophagi* strain K-2C isolated from a sheep ked (GenBank accession no. AY724770).

**Figure:**
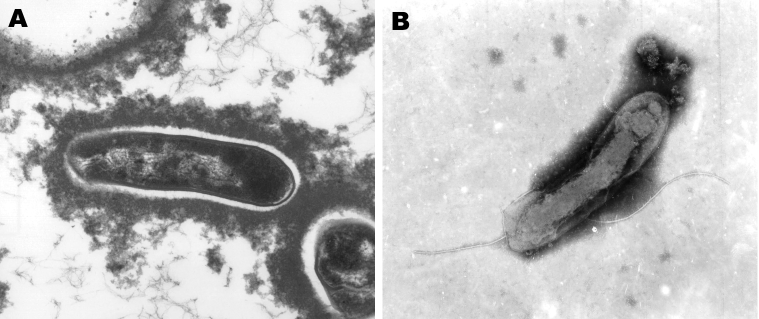
Transmission electron micrographs of Candidatus *Bartonella melophagi*–like isolate 05-HO-1 from a human (A) (image provided by the North Carolina State University–College of Veterinary Medicine Electron Microscopy Facility, Raleigh, NC, USA) and Candidatus *B. melophagi* isolate from a sheep ked (B) (image provided by V. Popov, University of Texas Medical Branch, Galveston, TX, USA). Magnification ×41,000 in A and ×62,700 in B.

## Conclusions

Based on 16S rRNA, citrate synthase and RNA polymerase B genes, and the 16S–23S ITS region, the bacteria detected in these woman was most likely Candidatus *B*. *melophagi*, which was recently isolated from sheep blood and sheep keds (*10*; M. Kosoy, unpub. data). ITS sequences were nearly identical to those of *Wolbachia melophagi* detected in a tick removed from sheep in Peru (*11*). Similar to electron micrographs of the *Bartonella* sp. isolated from sheep blood (*1*), no flagella were observed by transmission electron microscopy of the 05-HO-1 human isolate, whereas the sheep ked isolate contains flagella. Because both women had had frequent contact with numerous domestic and wild animals and potential insect vectors, the route of transmission is unknown.

The clinical relevance of Candidatus *B*. *melophagi* infection in these women remains to be established. Efforts to passage Candidatus *B*. *melophagi* in our laboratory and others (D.A. Bemis, *10*) have not been successful. Therefore, development of a serologic assay was not pursued. Nonspecific abnormalities, including difficulty sleeping, muscle weakness, joint pain, and facial tremors, have been reported in association with isolation of *B*. *henselae* and *B*. *vinsonii* subsp. *berkhoffii* (*2*,*12*). Pericardial or pleural effusions are infrequent complications of *B*. *henselae* infection in association with classical cat-scratch disease (*13*,*14*).

Before the report of Candidatus *B*. *melophagi* in commercial sheep blood sources in 2007 (*10*), sheep blood was used as a BAPGM supplement in our laboratory. With the exception of these 2 patients, Candidatus *B*. *melophagi* was never detected by PCR in >2,250 BAPGM enrichment blood cultures or subculture isolates obtained from animals or humans. In addition, Candidatus *B*. *melophagi* DNA was never amplified from >250 BAPGM uninoculated BAPGM enrichment control cultures, and bacterial colonies were never observed after subculture. Beginning in 2007, we also found that some batches of commercial sheep blood contained Candidatus *B*. *melophagi* DNA. Therefore, we no longer use blood as a BAPGM supplement. Recently, BAPGM was used to facilitate isolation of *B*. *tamiae* from human patients in Thailand (*7*), and another laboratory has published data supporting the utility of insect cell culture media for growing *Bartonella* spp. (*15*).
